# Multi-environment evaluation of rice genotypes: impact of weather and culm biochemical parameters against sheath blight infection

**DOI:** 10.3389/fpls.2023.1280321

**Published:** 2023-10-30

**Authors:** Siddharth Panda, Naveen kumar R., Lalitha Pavani S., Sangeetha Ganesan, Pawan Kumar Singh, Rameswar Prasad Sah, Padmakumar V., Hatanath Subudhi, Anumalla Mahender, Annamalai Anandan, Jauhar Ali

**Affiliations:** ^1^ Department of Genetics and Plant Breeding, Institute of Agricultural Sciences, Siksha 'O' Anushandhan (SOA) [Deemed to be University (DU)], Bhubaneswar, Odisha, India; ^2^ Division of Plant Pathology, School of Agricultural Sciences, Karunya Institute of Technology and Sciences, Coimbatore, Tamil Nadu, India; ^3^ Institute of Agricultural Sciences, Banaras Hindu University, Varanasi, Uttar Pradesh, India; ^4^ Crop Improvement Division, Indian Council of Agricultural Research (ICAR) - National Rice Research Institute (NRRI), Cuttack, India; ^5^ Plant Biosecurity Division, National Institute of Plant Health Management (NIPHM), Hyderabad, Telangana, India; ^6^ Indian Council of Agricultural Research (ICAR)-Indian Institute of Horticultural Research, Bengaluru, India; ^7^ International Livestock Research Institute (ILRI), Hyderabad, Telangana, India; ^8^ Rice Breeding Innovation Platform, International Rice Research Institute (IRRI), Los Baños, Laguna, Philippines; ^9^ Indian Council of Agricultural Research (ICAR)-Indian Institute of Seed Science, Bengaluru, India

**Keywords:** sheath blight, PDI, AUDPC, AMMI, GGE, MTSI, humidity, cellulose

## Abstract

**Introduction:**

Sheath blight caused by *Rhizoctonia solani* is one of the major diseases of rice, causing widespread crop losses. The use of semi-dwarf rice varieties in the ongoing nutrient-intensive rice cultivation system has further accentuated the incidence of the disease. An ideal solution to this problem would be identifying a stable sheath blight-tolerant genotype.

**Material and methods:**

A multi-environment evaluation of 32 rice genotypes against sheath blight infection was conducted over six seasons across two locations (Agricultural Research Farm, Institute of Agricultural Sciences, Banaras Hindu University (28.18° N, 38.03° E, and 75.5 masl), for four years during the wet seasons (*kharif*) from 2015 to 2018 and two seasons at the National Rice Research Institute (20°27’09” N, 85°55’57” E, 26 masl), Cuttack, Odisha, during the dry season (*rabi*) of 2019 and the *kharif* of 2019, including susceptible and resistant check. Percent disease index data were collected over 4 weeks (on the 7th, 14th, 21st, and 28th day after infection), along with data on other morphological and physiological traits.

**Result and discussion:**

The resistant genotypes across seasons were the ones with a higher hemicellulose content (13.93-14.64) and lower nitrogen content (1.10- 1.31) compared with the susceptible check Tapaswini (G32) (hemicellulose 12.96, nitrogen 1.38), which might explain the resistant reaction. Three different stability models—additive main effect and multiplicative interaction (AMMI), genotype + genotype x environment (GGE) biplot, and multi-trait stability index (MTSI)—were then used to identify the stable resistant genotypes across six seasons. The results obtained with all three models had common genotypes highlighted as stable and having a low area under the disease progress curve (AUDPC) values. The ideal stable genotypes with low disease incidence were IC 283139 (G19), Tetep (G28), IC 260917 (G4), and IC 277274 (G10), with AUDPC values of 658.91, 607.46, 479.69, and 547.94, respectively. Weather parameters such as temperature, rainfall, sunshine hours, and relative humidity were also noted daily. Relative humidity was positively correlated with the percent disease index.

## Introduction

Rice (*Oryza sativa* L., 2n=24), a member of *Poaceae*, is a staple crop in South Asian countries and provides 50% of the world’s calories (Pareja et al., 2011). It also creates 3.5 million person-days of employment, accounting for 10% of India’s agricultural GDP (Singh et al., 2021). Rice is used in various ways and is a good source of complex dietary carbohydrates, proteins, vitamins, and minerals (Roy et al., 2008; Durgadevi and Shetty, 2014; Rohman et al., 2014). India is the second-largest producer of rice after China, accounting for 20% of global rice production (Singh et al., 2019). India’s total rice production was 127.93 million metric tons in 2021-22 (www.indiastat.com). As an energy-giving food, rice consumption was as high as 103.5 million metric tons in India in the crop year 2020-21 (www.fao.org/worldfoodsituation).

The cultivation of rice is challenged by several biotic stresses, such as various diseases caused by fungi, bacteria, and viruses (Persaud et al., 2019). Among them, the fungal diseases blast (caused by *Magnaporthe oryzae*), sheath blight (caused by *Rhizoctonia solani*), and brown spot (caused by *Bipolaris oryzae*) are three of the prominent destructive diseases (Margani and Widadi, 2018). Sheath blight, a ubiquitous destructive soil-borne pathogen, is the most damaging disease in rice, second only to blast (Savary et al., 2006; Singh et al., 2019). High-yielding semi-dwarf cultivars with dense planting and high doses of nitrogenous fertilizers accentuate the incidence of sheath blight in rice. Its diverse host range and ability to remain dormant under unfavorable conditions make the pathogen more difficult to manage (Savary et al., 1995). The disease is also known as oriental sheath, leaf, sclerotial, or banded blight. The infection process of *R. solani* involves adhesion, penetration, and colonization. The fungal hyphae penetrate the stomata and produce lobate appressoria or infection cushions (Groth and Nowick, 1992; Neelam et al., 2017). The appressorium formation triggers the enzymatic degradation of cell wall components, promoting colonization, and this ultimately results in necrotic lesions, which later increase in length up to 2-3 cm and 1 cm in width, with bleached middle and purple-brown borders (Rush, 1992; Srinivas et al., 2013). These lesions coalesce and extend from leaf sheaths to leaf blades and panicles and eventually to other tillers (Groth and Nowick, 1992; Srinivas et al., 2013). Yield losses vary from 20% to 50% depending on the genotype, plant growth stage, and environmental conditions (Prasad et al., 2020). After harvesting the rice crop, sclerotia of *R. solani* from the infected plants remain in the soil and survive up to 3 years and can act as a source of disease infection for subsequent cropping seasons (Savary et al., 1995). The monocropping system can be a big challenge for farmers. To manage this, they may need to rotate crops with plants that are not susceptible to the same diseases. Additionally, the pathogen is difficult to manage due to its wide host range, long persistence of sclerotia, and ability to adapt to different environments (Singh et al., 2019). This pathogen often survives on alternate hosts during hostile conditions, making the disease very difficult to manage. Therefore, farmers must use fungicides to integrate cultural practices into their crop management (Persaud et al., 2019). However, the use of fungicides is known to pose various ecological problems, ultimately affecting human health (Goswami et al., 2019). Hence, developing resistant rice varieties is the best solution to this problem.

The major hurdle in developing a stable resistant donor for sheath blight is because of the complexity of the pathogen, resistance being a polygenic and variable response of the rice genotypes. Because of the non-availability of a stable resistant donor, breeding for rice tolerant to *R. solani* has been unsuccessful (Naveen kumar et al., 2022). The development of cultivars resistant to *R. solani* is a major thrust area for rice research and development across the globe, as this proposes an economically and environment-friendly and sound strategy for managing sheath blight (Dey et al., 2016). Genotypes with resistance reported thus far, such as Jasmine 85 and Teqing, eventually become susceptible (Dey et al., 2016; Naveen kumar et al., 2022). This may be due to the pathogen’s acquired resistance or the absence of a stable genotype against the pathogen *R. solani* (Naveen kumar et al., 2022). Additionally, the role of certain biochemical parameters such as cellulose, hemicellulose content, lignin content, etc., need to be studied to understand their relationship in the disease manifestation. Many recent works have attributed the disease development and severity to the phenolic content, lignin content, cellulose content, and hemicellulose content (Taheri and Hofte, 2007; Zhang et al., 2013; Hapsari and Poromarto, 2020).

Developing a cultivar for a trait such as disease resistance requires validation for stability across environments or seasons with varying weather attributions. The instability of the plant’s response in its disease reaction can be owed to genotype x environment (G x E) interaction. The role of G x E is essential for identifying and evaluating durable resistance sources (Singh et al., 2020). G x E interaction studies might give an insight into repeated field plots and greenhouse ratings in view of G x E. Several G x E studies have been documented for various diseases in rice, wheat, sorghum, and pearl millet crops (Mukherjee et al., 2013; Das et al., 2019; Persaud et al., 2019; Kirtphaiboon et al., 2021; Sankar et al., 2021). However, stability analysis and G x E for sheath blight of rice have not been widely studied, and few reports are available (Persaud et al., 2019). To assess G x E for resistance against sheath blight of rice, we evaluated rice genotypes across six seasons to assess the usefulness of testing environments and seasons. In order to explore how different genotypes perform differently under various environmental conditions, this study has used three different multivariate stability models. Biplots are frequently used to illustrate interaction patterns, identify similarly stable genotypes across environments, and illustrate the links between genotype (G), environment (E), and G x E. Among the biplot models, the most popular are AMMI (additive main effects and multiplicative interaction) and GGE (genotype + genotype x environment) biplots. There have been many reports of stability analysis based on these models (Silva et al., 2011; Thangavel et al., 2011; Lakew et al., 2017; Persaud and Saravanakumar, 2018; Sandhu et al., 2019; Yue et al., 2022). A new multivariate stability analysis model, the multi-trait stability index (MTSI), is also employed in this study. The need to consider multiple traits simultaneously to identify a stable genotype necessitated the development of statistical procedures such as MTSI (Olivoto et al., 2019). It is founded on factor analysis, and the factorial scores for each ideotype are constructed in accordance with desirable and undesirable traits. In order to enable accession ranking, a spatial probability is calculated based on accession-ideotype distance. Since the accession with the lowest MTSI is closest to the ideal, it performs better on average and is more stable across all of the examined factors. However, there are limited reports on the study of the stability of sheath blight-resistant lines. The knowledge gathered from this inquiry will provide benefits in choosing stable resistant genotypes for the intended growing environments, as well as in understanding the influence of G x E interaction and helping to discover the environmental elements responsible for variation in the amount of resistance. The inferences from each stability model can also compare their robustness and the results’ similarities. Identifying stable genotypes will help develop tolerant varieties through marker-assisted breeding programs and further genomic dissection of these resistant lines.

## Materials and methods

### Planting materials

A total of 32 rice (*Oryza sativa* L.) genotypes (**Supplementary Table 1**), which consisted of 26 popular varieties and landraces along with susceptible (Pusa Basmati-1 and Tapaswini) and resistant (Tetep, Jasmine 85, and Teqing) checks, were selected for this study. These rice genotypes were obtained from the Department of Genetics and Plant Breeding, Institute of Agricultural Sciences, Banaras Hindu University, Varanasi, Uttar Pradesh, and ICAR-National Rice Research Institute (NRRI), Cuttack, Odisha. To evaluate these genotypes against *R. solani*, a field trial was managed at the Agricultural Research Farm, Institute of Agricultural Sciences, Banaras Hindu University (28.18° N, 38.03° E, and 75.5 masl), Varanasi, for 4 years during the wet season (*kharif*) from 2015 to 2018 and two seasons at NRRI (20°27’09” N, 85°55’57” E, 26 masl), Cuttack, Odisha, during the dry season (*rabi*) of 2019 and the *kharif* of 2019 (**Table 1**). The nursery beds were prepared following the recommended package of practices. The seeds of each genotype were sown in a row length of 50 cm. Thereafter, the beds were covered with a thin film of water. Irrigation was done at regular intervals to maintain sufficient moisture in the nursery beds. The rice seedlings (25-30 days after sowing) were uprooted carefully from the beds and transplanted into the main field, which was divided into blocks. A row of 2.0 m and plant-to-plant spacing of 20 cm was maintained for each genotype. The experiment was performed in a randomized block design with three biological replications.

**Table 1:** Temperature, the status of rainfall, relative humidity, soil properties, latitude, altitude and code for each environment.

Season/environment	Maximum Temperature (°C)	Minimum Temperature (°C)	Rainfall (mm)	Sunshine hours	RH (morning) (%)	RH (Evening) (%)	PDI MEAN
*kharif* 2015 (E1)	33.4	22.1	0.0	8.4	83.0	54.4	25.21
*kharif* 2016 (E2)	31.9	25.6	6.9	4.2	87.3	77.7	28.62
*kharif* 2017 (E3)	33.5	25.5	1.0	6.4	89.8	68.6	20.19
*kharif* 2018 (E4)	33.0	20.2	0.0	NA	86.3	53.1	28.62
*kharif* 2019 (E5)	31.1	24.0	26.6	5.4	92.3	71.8	25.05
Rabi 2019 (E6)	36.6	25.2	7.8	6.3	87.4	60.5	28.08

NA, not available.

### Pathogen inoculation

A highly virulent strain (MTCC12227) of *R. solani* belonging to the AG-1 IA group collected from the Department of Mycology and Plant Pathology, Institute of Agricultural Sciences, BHU, was used to inoculate the rice genotypes for screening study across six seasons. This isolate was previously reported as a highly virulent isolate by pathogenicity test in our previous study (Goswami et al., 2019). The fungus was grown on a PDA medium at 25 ± 2°C for 72-96 h. Five plants were selected from the middle for inoculation when they were 45 days old. The inoculum was placed beneath the third leaf sheath from the top by placing the 3- to 4-day-old sclerotia beneath the rice leaf sheath and tying it with wet cotton to provide sufficient moisture for the development of the fungus (Singh et al., 2002).

### Disease assessment

Disease severity in each genotype was recorded at weekly intervals, at 7, 14, 21, and 28 days after inoculation based on relative lesion height (RLH). This parameter was used to obtain the exact percentage of infection and, using this RLH, a disease scale (0-9) was given to each plant. RLH was calculated using the following formula given by Sharma and Teng (1990):


$$RLH=\frac{Maximum\;height\;at\;which\;lesion\;appears}{Plant\;height\;\left(cm\right)}\times 100$$

Disease scoring (0-9 scale) was done using a standard evaluation system (SES) to measure disease severity, where 0 = free from infection, 1 = lesion limited to the lower 20% of the plant height, 3 = 20-30%, 5 = 31-45%, 7 = 46-65%, and 9 = more than 65% (IRRI (International Rice Research Institute), 2013). Using a standardized evaluation system can help to maintain a uniform and replicable response from the test genotypes. Percent disease index (PDI) was calculated using the formula reported by Wheeler (1969):


$$PDI=\frac{Sum\;of\;all\;ratings}{Total\;no.\;of\;observations\times Maximum\;rating\;scale}\times 100$$

However, the area under the disease progress curve (AUDPC) was calculated based on the disease severity percentage of each disease score taken four times using the formula given by Shaner and Finney (1977):


$$AUDPC=\sum_{i=1}^{n}\left\{\left[\left(Xi+1\;+\;Xi\right)\right]/2\;x\;\left(ti+1\;–\;ti\right)\right\}$$

where X_i_ is the disease index expressed as a proportion at the i^th^ observation, the time (days after planting) at the i^th^ observation, and n is the total number of observations. PDI was calculated to know the prevalence of the disease, while the AUDPC was calculated to determine disease progress from the date of inoculation and the difference between genotypes in terms of their disease response.

Other morphological traits, such as plant height (cm), tiller number, panicle length (cm), flag-leaf length (cm), flag-leaf width (cm), internodal length (cm), culm thickness (mm), ligule color, ligule shape, auricle color, basal leaf sheath color, and apiculus color, were measured during the reproductive phase following the SES for rice developed by the International Rice Research Institute, Philippines (IRRI (International Rice Research Institute), 2013). Straw was harvested from each replication, ground, and filtered through 1-mm mesh. The sample collection and estimation of quality were carried out using the method used by Subudhi et al. (2020). The straw samples were evaluated at the International Livestock Research Institute, Hyderabad center. The straw was evaluated for cell wall components such as dry matter (DM), ash content (AC), nitrogen (N), lignin (Li), silica (Si), digestibility (*in vitro* organic matter digestibility, IVOMD), cellulose (Cl), hemicellulose (HC), and digestibility (Di). Straw samples were analyzed using calibrated near-infrared spectroscopy (NIRS) (FOSS Forage Analyzer 6500 with software WinISI II). Details of the calibration and optimization of NIRS for straw quality estimation were published by Subudhi et al. (2020).

Analysis of variance and variability parameters were calculated using “Prog for variability ver 01.12.2020” (Manivannan, 2014) and descriptive statistics using the variability package in R (Gopinath et al., 2021). Principal component analysis (PCA) was carried out using the FactoMine R package (Lê et al., 2008), and correlation among all the studied traits was analyzed using the corrplot functions from the corrplot package (Wei and Simko, 2021) in R 4.0.3. Variance components were estimated, like heritability, heritability of genotypic mean (h^2^_mg_), genotypic coefficient of variation (CV_g_), and relative coefficient of variation (CV_r_), following REstricted Maximum Likelihood (REML) using the expectation-maximum algorithm (Dempster et al., 1977). 

The AMMI model was applied, with additive effects for the 32 rice genotypes (G), six seasons of testing (environments = E), and multiplicative term for G x E interactions. It makes use of the standard ANOVA procedure to separate the additive variance from the multiplicative variance (genotype x environment interaction). Then, it uses a multiplicative procedure (PCA) to extract the pattern from the G x E portion of the ANOVA. The result is the least square analysis, which, with a further graphical representation of the numerical results (biplot analysis), often allows a straightforward interpretation of the underlying causes of G x E interactions. The mathematical statement of the hybrid model is as follows:


$${\;}_{\text{Yge}}=\text{ μ }+\;{\text{α}}_{\text{g }+}{\text{β}}_{\text{e }+}\sum^{\text{N}}\lambda_{\text{n}}{\text{δ}}_{\text{gn}}{\text{η}}_{\text{en}}+\;{\text{θ}}_{\text{ge}}$$

where g = genotype, e = environment, Y_ge_ = disease index of genotype ‘g’ in environment ‘e’, μ = grand mean, α_g_ = the genotype mean deviation, β_e_ = the environment mean deviation, N = the number of IPCA (interaction principal component axis) retained in the model, λ_n_ = the eigenvalue for IPCA axis ‘n’, δ_gn_ = the genotype PCA scores for the IPCA axis ‘n’, η_en_ = the environment PCA scores for IPCA axis ‘n’, and θ_ge_ = the residuals. The GGE model was calculated on the basis of the following formula:


$${\text{Y}}_{\text{ge}}=\text{ μ }+\;\beta_{\text{e}}+\;\sum^{\text{N}}\lambda_{\text{n}}{\text{δ}}_{\text{gn}}{\text{η}}_{\text{en}}+\;{\text{θ}}_{\text{ge}}$$

To estimate the multi-trait stability index (MTSI) (30), the following equation was used:


$${\text{MTSI}}_{\text{i}}=[\sum_{\text{j}=1}^{\text{f}}.{\left(\text{Fij}-\text{Fj}\right)}^{2}]0.5$$

where MTSI_i_ is the multi-trait stability index for the i^th^ genotype, F_ij_ is the j^th^ score of the i^th^ genotype, and F_j_ is the j^th^ score of the ideotype. Therefore, the genotype with the lowest MTSI is closer to the ideotype and has a high mean performance and stability for all of the variables studied. Multi-environment trial data stability analyses using MTSI and WAASB indices were conducted using the *metan* package (Olivoto and Lúcio, 2020) of R 4.0.3 software.

## Results

The study focused on measuring disease incidence parameters along with morphological, physiological, and biochemical attributes; identifying associations with disease symptom development; and studying the stability of sheath blight resistance in the genotypes tested. The mean performance of the traits was studied from data collected across six seasons (**Supplementary Table 1**). The mean plant height was 105.53 cm, the average tiller number was 10.45, and the mean panicle length was 23 cm. The mean value of flag-leaf length was 28.46 cm, the mean flag-leaf width was 1.28 cm, and the mean internodal length was 28.81 cm. All of these traits followed a normal distribution, as verified by Kolmogorov–Smirnov (K-S) and K-S modified tests.

The biochemical parameters of the stem, such as dry matter content, nitrogen content, neutral detergent fiber, acid detergent fiber, silica content, cellulose, hemicellulose, and digestibility, followed a normal distribution. Ash content and acid detergent lignin did not follow a normal distribution, as per the K-S modified test. The mean nitrogen was 1.23, the mean hemicellulose content was 12.65, and the mean cellulose content was 46.63. The other studied traits such as culm thickness, ligule color, auricle color, ligule shape, basal leaf sheath color, and apiculus color didn’t follow a normal distribution as per both the K-S test and K-S modified test.

The disease reaction-measuring traits PDI 7th, PDI 14th, PDI 21st, and PDI 28th day had an increasing trend, with mean values of 12.08, 17.95, 31.72, and 42.23, respectively. The mean PDI over the 4 weeks was 25.94, with a range of 16.34 to 25.66, and the mean AUDPC value was 748.71, ranging from 479.68 to 1261.42. PDI 14th day and PDI 28th day did not follow a normal distribution and were rejected by the K-S modified test. In the case of PDI 14th day, the data were skewed toward the right (2.19), with a kurtosis peak at 7.55 (**Supplementary Table 2**). The skewness of the AUDPC was 1.59 (skewed right) and the kurtosis peak was at 6.13. The CV for all the disease (as mentioned above) reaction-measuring traits was within 20%, except for PDI 14th day, which had a Coefficient of variation (CV) of 28.79%. This indicates the variability in the intensity of infection by the 14th day across locations/seasons. The mean AUDPC was 748.71 across locations, while it was highest in 2019 *rabi* (830.14) and lowest in 2017 *kharif* (590.87) (**Table 2**). The mean PDI on the 7th day was 12.08 across seasons. The rate of increase of infection was highest from the 14th day to the 21st day. The PDI mean of all four observations taken on the 7th, 14th, 21st, and 28th day was 25.94. The highest PDI mean was in 2018 *kharif* (28.61) and the lowest was in 2017 (20.19). The CV was within 20% for all the traits except for tiller number and PDI on the 14th day. The K-S test proved that the data observed followed a normal distribution for all the traits.

**Table 2:** Mean AUDPC values of 32 rice genotypes across six different seasons, standard deviation (SD), and coefficient of variation (CV).

Genotype	Environment/season						Mean	SD	CV
	2015	2016	2017	2018	2019 *rabi*	2019 *kharif*			
IC 277237 (G1)	697.52	918.51	437.50	700.00	1008.56	934.06	782.69	212.43	27.14
IC 256613 (G2)	707.28	761.52	460.83	700.00	699.65	624.17	658.91	106.45	16.16
IC 256616 (G3)	744.06	841.74	761.25	1061.67	1052.40	77.77	756.48	360.22	47.62
IC 260917 (G4)	600.88	657.94	430.21	670.83	706.19	578.69	607.46	98.59	16.23
IC 264141 (G5)	763.35	776.73	801.11	1081.11	890.03	490.58	800.49	192.23	24.01
IC 274377 (G6)	730.88	698.11	488.06	855.56	889.61	1245.05	817.88	252.88	30.92
IC 274408 (G7)	597.69	739.04	459.86	696.11	772.47	760.22	670.90	121.22	18.07
IC 277248 (G8)	788.14	1060.33	476.39	855.56	864.61	575.56	770.10	212.09	27.54
IC 277261 (G9)	704.76	781.13	437.50	700.00	701.67	563.89	648.16	124.83	19.26
IC 277274 (G10)	694.10	698.60	449.17	700.00	685.69	500.69	621.38	114.71	18.46
IC 277284 (G11)	654.60	824.65	511.39	855.56	801.21	591.11	706.42	140.87	19.94
IC 277290 (G12)	717.68	775.81	505.56	738.89	762.69	831.25	721.98	112.82	15.63
IC 277332 (G13)	529.20	756.43	595.00	816.67	755.44	873.06	720.96	132.19	18.34
IC 279355 (G14)	713.48	837.66	460.83	700.00	718.28	655.90	681.03	123.65	18.16
IC 280478 (G15)	589.40	700.60	587.22	972.22	755.72	752.50	726.28	141.97	19.55
IC 280504 (G16)	702.24	856.81	797.22	832.22	1054.52	777.85	836.81	119.14	14.24
IC 280564 (G17)	688.52	903.52	624.17	980.00	902.87	605.09	784.03	163.40	20.84
IC 281508 (G18)	786.97	891.93	511.39	855.56	857.22	917.78	803.47	149.72	18.63
IC 283139 (G19)	476.96	519.63	402.50	653.33	475.69	350.00	479.69	104.45	21.77
IC 282460 (G20)	728.25	855.89	733.06	948.89	923.78	991.67	863.59	112.00	12.97
IC 282812 (G21)	603.54	844.54	447.22	505.56	872.08	1239.26	752.03	295.32	39.27
IC 282815 (G22)	707.88	853.94	666.94	544.44	778.27	643.60	699.18	108.03	15.45
IC 283204 (G23)	638.27	731.23	505.56	972.22	833.41	637.78	719.75	164.94	22.92
IC 277267 (G24)	610.21	983.94	446.25	688.33	851.69	521.11	683.59	203.28	29.74
IC 277275 (G25)	581.84	1093.15	597.43	686.39	1061.21	768.86	798.14	226.56	28.39
IC 283206 (G26)	753.98	957.45	453.06	762.22	836.50	668.89	738.68	170.10	23.03
CO 39 (G27)	869.70	884.39	698.06	1088.89	779.23	627.41	824.61	162.74	19.73
Tetep (G28)	663.10	482.01	489.03	602.78	568.74	482.01	547.94	75.98	13.87
Pusa Basmati-1 (G29)	853.14	981.85	634.38	1312.50	1023.50	981.85	964.54	222.07	23.02
Jasmine-85 (G30)	655.87	561.26	715.56	738.89	672.69	561.26	650.92	75.49	11.60
Teqing (G31)	871.33	916.27	820.56	676.67	955.49	916.27	859.43	100.74	11.72
Tapaswini (G32)	1297.65	1124.39	1503.70	1464.81	1053.59	1124.39	1261.42	190.86	15.13

### Association between traits and principal component analysis

**Figure 1:**
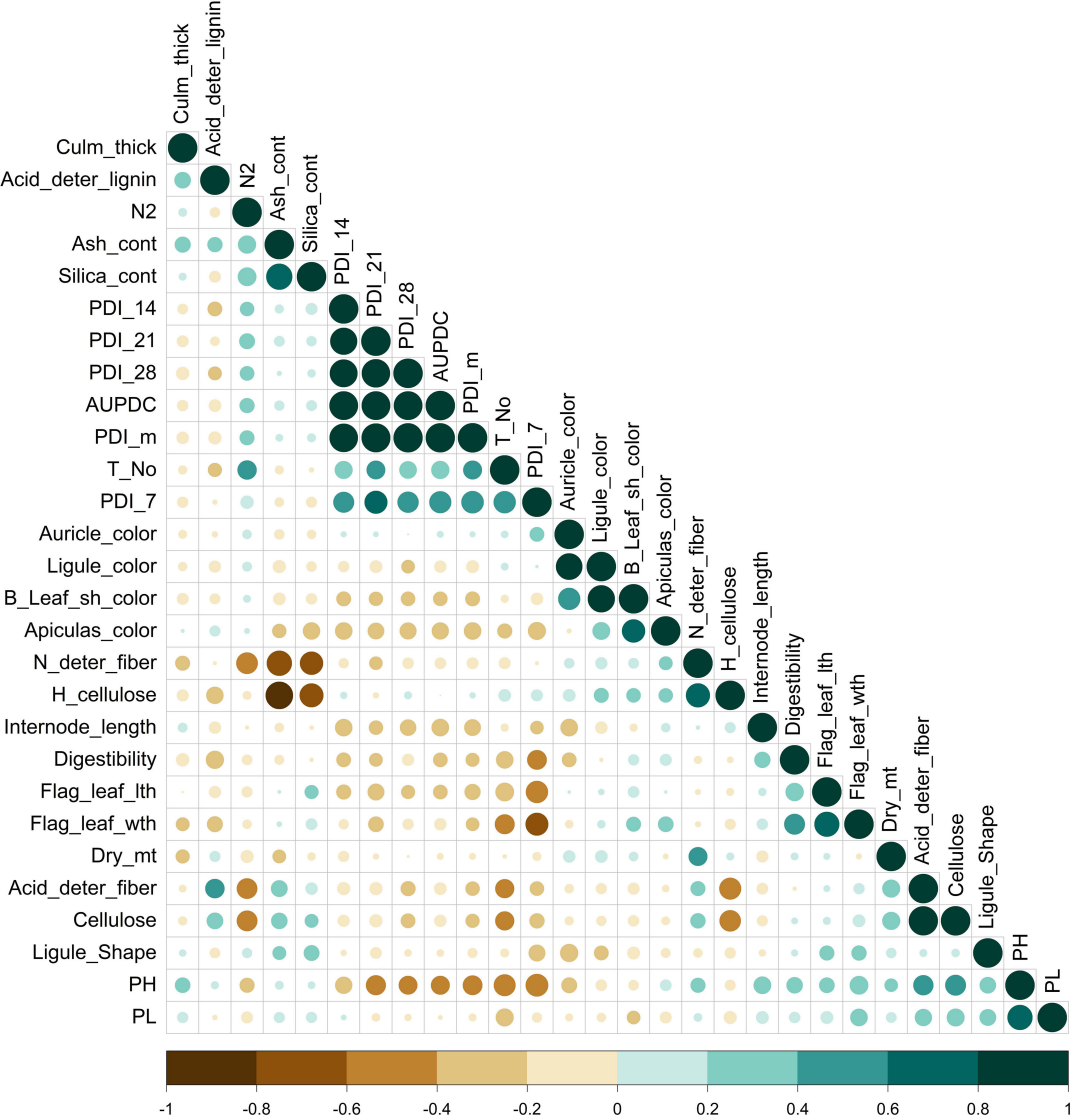
Pearson correlation for morphological, physiological, and biochemical parameters related to sheath blight disease resistance in rice of pooled mean over season. Color (green, positive correlation; brown, negative correlation) intensity and circle size are proportional to the correlation coefficient. (N2, nitrogen content; Dry_mt, dry matter; PH, plant height; Leaf_Sh-color, leaf sheath color; deter_fiber, detergent fiber; H_cellulose, hemicellulsose; Flag_leaf_lth, flag-leaf length; Flag_leaf_wth, flag-leaf width; T_No, tiller number; PL, panicle length; PDI_7, 14, 21, and 28 and m, percent disease index recorded on 7th, 14th, 21st, and 28th days and the mean, respectively).

Correlation analysis among the studied traits showed that the PDI values scored on the 7th day (0.57), 14th day (0.94), 21st day (0.95), and 28th day (0.95) had a significant positive correlation with AUDPC and a negative correlation with plant height (-0.40) (**Figure 1**). Correlation analysis suggested an association of AUDPC with tiller number (0.37), internodal length (-0.34), and PDI mean (0.98). Flag-leaf length and flag-leaf width significantly negatively correlated with PDI 7th day (i.e., -0.56 and -0.60, respectively) (**Supplementary Table 3**). There was an absence of any significant association of plant disease estimating traits with traits other than those mentioned above. The weather parameters, namely maximum temperature, minimum temperature, rainfall, sunshine hours, and relative humidity (RH) in the morning and evening were analyzed for study correlation with the PDI mean (**Supplementary Table 4**). The PDI mean had a significant negative correlation for minimum temperature (-0.91) and maximum temperature (-0.54). A positive correlation of relative humidity was recorded in the morning with the PDI mean (0.74).

**Figure 2:**
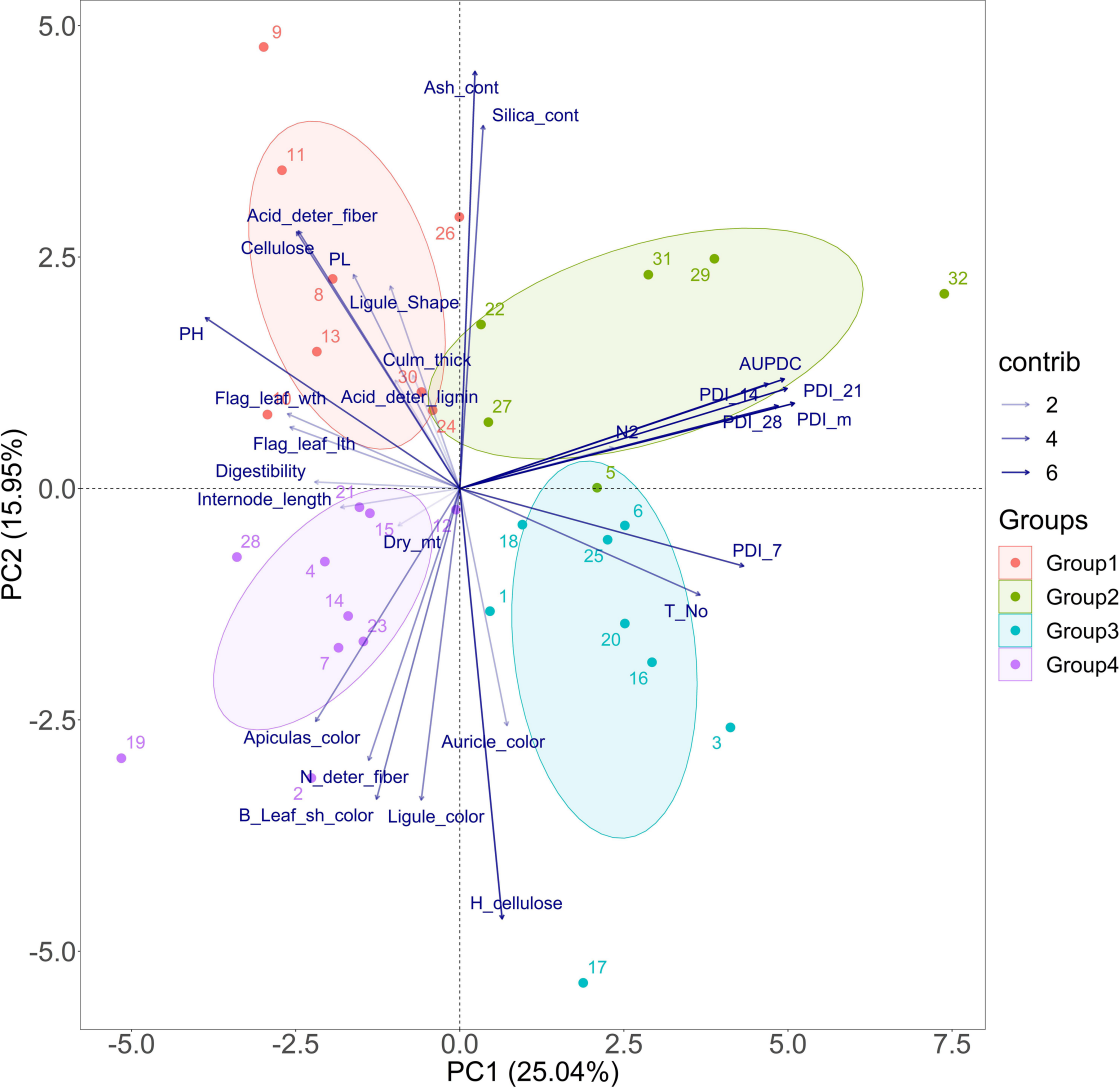
PCA for sheath blight disease resistance-related traits of the pooled mean over seasons explained by two axes. Together, the two PC axes explained 41% of the total variation. The transparency of the vector indicates the contribution to the variation in the dataset, ranging from 2% (lightest) to 6% (darkest). The direction and length of the vector represent the trait contribution to the first two components of the PCA. Group 2 (solid algae tone) and Group 3 (solid cyan tone) were found to be susceptible to sheath blight disease, with high AUDPC values. Group 1 (solid red tone) was moderately resistant and Group 4 was resistant against sheath blight (solid purple tone), sharing low values for AUDPC and PDI mean. The closed circle dots represent 32 genotypes used in the study. The genotype and corresponding numbers can be seen in **Supplementary Table 1**. Resistant check (Tetep)-28, Susceptible check (Tapaswini)- 32. N2, nitrogen content; Dry_mt, dry matter; PH, plant height; Leaf_Sh-color, leaf sheath color; deter_fiber, detergent fiber; H_cellulose, hemicellulsose; Flag_leaf_lth, flag-leaf length; Flag_leaf_wth, flag-leaf width, T_No, tiller number; PL, panicle length; PDI_7, 14, 21, and 28 and m, percent disease index recorded on 7th, 14th, 21st, and 28th days and the mean, respectively).

Principal component analysis was done to visualize the highest contributing traits to the variability recorded in the population. The eigenvalues of nine PCs were more than unity (**Supplementary Table 5**). The PDI mean, PDI 21st day, and AUDPC traits had the highest contribution to the variability in the whole population. Apart from the disease measuring traits, tiller number also contributed in a major way to the variability existing in the population. The susceptible check Tapaswini is placed in the first quadrant (group 2), opposite the one with the tolerant check Tetep (group 4) (**Figure 2**). Thus, the cluster of genotypes in the quadrant with Tapaswini can be regarded as susceptible, with high values of AUDPC, PDI 14th day, PDI 21st day, PDI 28th day, and PDI mean (**Figure 2**). The cluster formed with Tetep (the tolerant check) consists of test genotypes such as IC 283139 (G19), IC 260917 (G4), IC 256613 (G2), and IC 279355 (G14), among others, which have low mean values for AUDPC.

### G x E interaction and stability analysis

The results from the variability studies, correlation, and PCA helped in identifying the important traits that play a role in disease development in rice plants; these were AUDPC, plant height, tiller number, panicle length, PDI 7th day, PDI 14th day, PDI 21st day, PDI 28th day, and PDI mean. The 32 rice genotypes, including the resistant and susceptible check, were evaluated via the AMMI model to identify the stable sheath blight-resistant genotypes. The pooled ANOVA of sheath blight reaction on the test genotypes was analyzed via AUDPC values (calculated from the PDI values on the 7th, 14th, 21st, and 28th day). The combined analysis of the variance of the test genotypes across the six seasons highlighted variations due to genotype (G) and genotype x environment (G x E) as significant (P<0.001) (**Table 3**). The effect of G and G x E was significant, while the effect of environment (E) was non-significant. The relative contribution was calculated for each source: the environment factor contributed 11.02%, the G x E factor contributed 24.67%, whereas the genotype factor contributed 25.67% of the total sum of squares. In AMMI analysis, the variance of G x E interaction was partitioned into five principal components (PC), of which PC1, PC2, and PC3 were significant (P<0.01). PC1 and PC2 contributed significantly to the total variability (i.e., 53% and 23%, respectively) and PC3 explained a variation of 13.80%. The coefficient of determination (R^2^_ge_) value was low to moderate for all the traits studied; the highest was 0.31 for AUDPC (**Table 4**). Heritability was moderate for PDI 7th day and plant height, whereas it was low for all other traits. The genotypic selection accuracy ranged from 0.67 (PDI 7th day) to 0.94 (plant height) and 0.92 (PDI mean). The highest CVg was recorded for PDI 14th day, whereas the lowest was for PDI 7th day (**Table 4**).

**Table 3:** Analysis of variation of AMMI for 32 rice genotypes against sheath blight infection across six seasons.

Source	DF	SS	MSS	F value	PR (>F)	Contribution to variation (%)
Environment	5	2900639	580128	4.13	0.0568	11.03
Replication (ENV)	6	842941	140490	9.24	7.33E-09	3.20
Genotype (GEN)	31	6754793	217897	14.33	1.12E-34	25.68
GEN : ENV	155	6489661	41869	2.75	2.98E-11	24.67
PC1	35	3438420	98241	6.46	0	53.00
PC2	33	1490867	45178	2.97	0	23.00
PC3	31	893325	28817	1.90	0.005	13.80
PC4	29	477570	16468	1.08	0.366	7.40
PC5	27	189480	7018	0.46	0.990	2.90
Residuals	186	2827695	15203			
Total	538	26305390	48895			

SS, sum of squares; MSS, mean sum of squares.

**Table 4:** Genetic parameters for nine traits of 32 rice genotypes in response to sheath blight infection across six seasons.

Traits	σ^2^_p_	Heritability	r^2^_GEI_	h^2^_mg_	Accuracy	r_ge_	CV_g_	CV_r_	CV ratio
AUDPC	43205	0.34	0.31	0.808	0.899	0.467	16.2	16.5	0.982
Plant height	756	0.50	0.30	0.883	0.94	0.598	18.5	11.7	1.58
Tiller number	15.9	0.42	0.18	0.868	0.932	0.302	24.7	24.4	1.01
Panicle length	15.8	0.40	0.23	0.852	0.923	0.377	10.9	10.6	1.03
PDI_7th day	6.34	0.07	0	0.455	0.674	0	5.31	20.1	0.264
PDI_14th day	63.8	0.35	0.15	0.842	0.918	0.226	26.4	31.5	0.839
PDI_21st day	80.5	0.31	0.30	0.795	0.892	0.407	15.8	18.1	0.875
PDI_28th day	140	0.35	0.17	0.834	0.913	0.265	16.5	19.4	0.85
PDI_mean	44	0.39	0.23	0.848	0.921	0.383	16	15.7	1.02

σ^2^_p_, phenotypic variance; r^2^_GEI_, the coefficient of determination for GEI effects; h^2^_mg_, heritability of the genotypic mean, r_ge_, association among genotypic values across environments CV_g_ and CV_r_ are the genotypic and variation coefficients of variation, respectively.

### AMMI biplots

**Figure 3:**
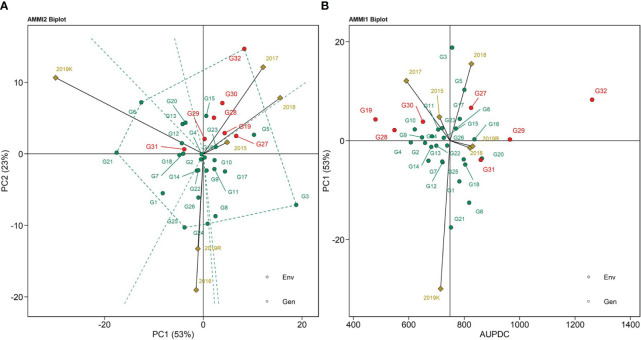
**(A)** AMMI 1 biplot for AUDPC values using the genotypic and environmental scores, **(B)** AMMI 2 biplot for AUDPC values showing the interaction of PC1 vs. PC2 loadings of 32 rice genotypes (G) and six environments.

The AMMI 1 biplot represents sheath blight AUDPC values and the abscissa shows differences in main effects and the ordinate differences in interaction patterns (**Figure 3A**). IPCA scores for both genotype and environment main effect were plotted against the AUDPC values for sheath blight. The AMMI 1 biplot captures 53% of the variability due to AUDPC in the test population (**Figure 3A**). Tapaswini (G32) had the highest mean AUDPC value (undesirable), whereas Tetep (G28), IC 277274 (G10), and IC 283139 (G19) are the genotypes that displayed lower values of AUDPC. The 2016 and 2019 *rabi* vectors were placed close to zero, showing a high positive association. The AMMI 2 biplot is represented in **Figure 3B**, with the environmental scores drawn to the origin and the distant points all connected to form a polygon. The polygon had vertices with G6 (IC 274377) representing 2019 *kharif*; G32 (Tapaswini) representing 2017, 2018, and 2015; and G3 (IC 256616), G25 (IC 277275), and G21 (IC 282812) representing 2016 and 2019 *rabi* (**Figure 3B**). The 2015 season had the shortest vector; environments with short vector lengths exerted weak interactive force. Hence, the highest interactions appeared in 2019 *kharif* and 2016. PC1 and PC2 explained G x E interaction accounting for 76% of the total variability in the AMMI 2 biplot. The angle between the vectors of 2015, 2017, and 2018 was less and represented a positive association among them. But the angles between 2018 and 2019 *rabi*, 2018 and 2016, and 2019 *rabi* and 2019 *kharif* displayed a negative association. The genotypes lying closer to the point of origin (preferably on the right-hand side) showcase stability, with less variation in AUDPC value and less interaction with the environment. Such genotypes closer to the origin are G22 (IC 282815), G15 (IC 280478), G4 (IC 260917), and G14 (IC 279355). However, the stability of these genotypes is only useful if the AUDPC values are reliable and lower than those of the susceptible check.

### GGE biplot pattern for elucidation of multivariate analysis

In the assessment of multi-environment trials, the effects of genotype and G x E interactions are the major sources of variation (Yan et al., 2000). An ideal genotype is selected based on its mean performance and level of interaction with its environment. The GGE biplots were created to identify the most tolerant line out of the genotypes tested against sheath blight infection. The biplots are constructed using two axes: PC1 denotes the magnitude of the trait under study and PC2 measures the stability. Three different graphical patterns can infer the GGE biplot: (a) “which-won-where” pattern, (b) mean vs. stability performance, and (c) discriminativeness vs. representativeness of the test environment for genotype evaluation.

#### Which-won-where model

**Figure 4:**
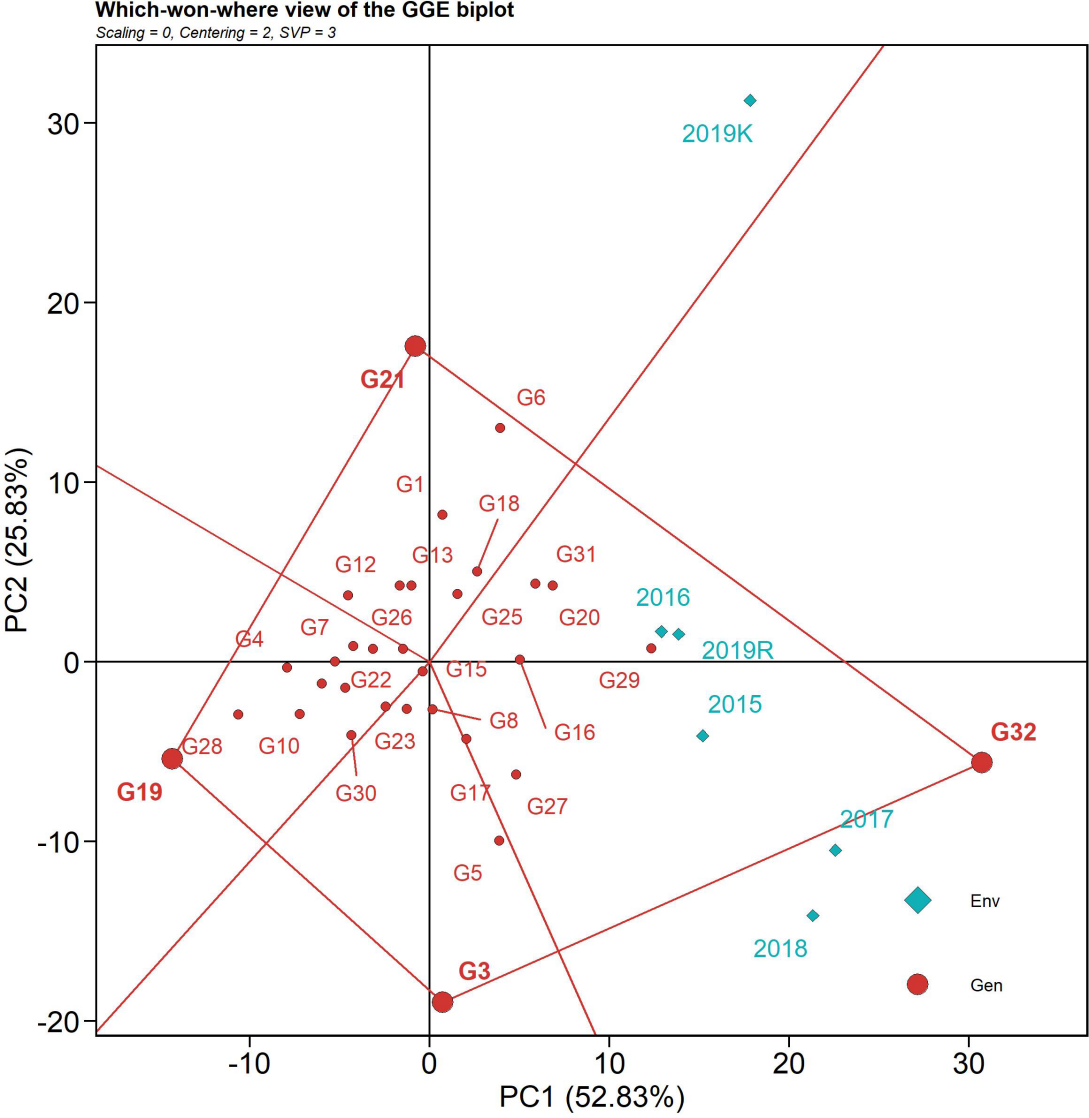
The “which-won-where” polygon view of the GGE biplot based on the G x E data of the 32 rice genotypes for AUDPC value against sheath blight of rice. The data were not scaled (“Scaling = 0”) and were environment-centered (“Centering = 2”). The biplot was based on genotype-focused singular value partitioning (“SVP = 1”) and, therefore, is appropriate for visualizing genotype similarities. It explained 78.6% of the total G + GE for the subset.

The polygon view of the GGE biplot, which depicts the “which-won-where” pattern of a multi-environment dataset, is the most efficient and concise method of summarizing the genotype and G x E interaction (**Figure 4**) (Bishwas et al., 2021). The environmental indicators were placed in two sections of the biplot for AUDPC, with different genotypes winning in each segment. Based on the 32 genotypes and six environments, the GGE biplot was divided into four fan-shaped sections. Five of the six seasons (2015, 2016, 2107, 2018, and 2019 *rabi*) were clustered within one sector, whereas the 2019 *kharif* was placed in an adjacent sector. Lower AUDPC values were observed on the opposite side of the sector, with genotype IC 283139 (G19) at the vertex. IC 283139 (G19) had a low AUDPC value (479.69) but was placed away from the origin, thus being deemed inconsistent in its reaction over the seasons (with a CV of 21.77) vis-à-vis those that were placed in proximity to the origin. IC 260917 (G4), Tetep (G28), IC 277274 (G10), and IC 256613 (G2) were nearer to the origin, indicating consistency in reaction over the seasons along with a low AUDPC value. Additionally, Tetep (G28), IC 260917 (G4), and IC 277274 (G10) were nearer to the vertex with IC 283139 (G19) and displayed lower AUDPC values (547.94 (CV 13.87), 607.46 (CV 16.23), and 621.38 (CV 18.46), respectively) and comparatively more stability. Moreover, the nitrogen, cellulose, and hemicellulose levels in these genotypes varied from 1.10 to 1.28, 45.37 to 48.45, and 11.53 to 14.64, respectively, which might explain the source of resistance. On the contrary, Tapaswini (the susceptible check) (G32) was located in the vertex of the sector exactly opposite IC 283139, with the highest AUDPC value for sheath blight. The nitrogen, cellulose, and hemicellulose levels in Tapaswini were 1.32, 45.42, and 12.96, respectively.

#### Mean vs. stability: identifying stable genotypes with desirable trait expression (GGE biplots)

**Figure 5:**
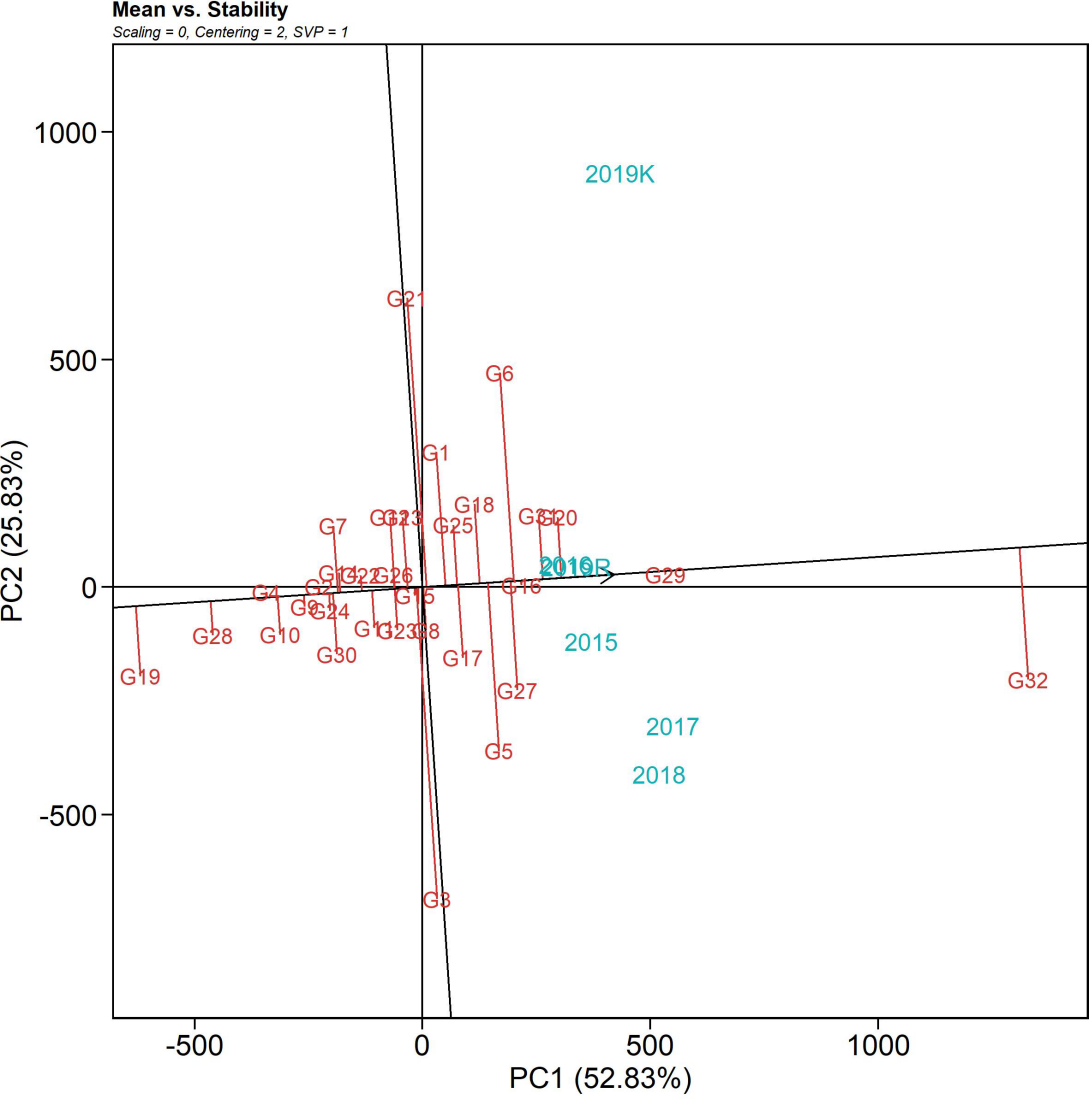
The mean vs. stability pattern of GGE biplot illustrating the interaction effect of 32 rice genotypes under six seasons in two locations for AUDPC values against sheath blight of rice. The data were not scaled (“Scaling = 0”) and were environment-centered (“Centering = 2”). The biplot was based on genotype-focused singular value partitioning (“SVP = 1”) and, therefore, is appropriate for visualizing genotype similarities. It explained 78.6% of the total G + GE for the subset.

The mean vs. stability biplot of the genotypes over six seasons is graphically presented in **Figure 5** through an “average environment coordinates” view of the biplot. The single arrow line traversing the biplot origin is the abscissa, which indicates the AUDPC value, and the line perpendicular to the abscissa at the origin is the ordinate. The length of the abscissa represents the AUDPC value (i.e., high disease incidence on the right-hand side of the origin and vice versa). The length of the ordinates on the abscissa estimates the corresponding GEI (i.e., if the length is less, then it corresponds to higher stability). The best-performing genotypes would be those with the lowest AUDPC values (with higher negative projections) and high stability (projection of genotypes on the abscissa that are close to 0). In the AUDPC mean vs. stability biplot, PC1 explained 52.83%, and PC2 explained 25.83% of the variation due to the G + G x E variation. IC 283139 (G19) is a desirable genotype with a lower disease incidence score than the tolerant check, Tetep (G28), and above-average stability. Its AUDPC value across seasons was 479.69, with a CV of 21.77%. The genotypes plotted close to IC 283139 (G19) can be treated as desirable in consideration of their Euclidean distances. The other test genotypes, namely, IC 260917 (G4), IC 277274 (G10), IC 256613 (G2), and IC 277261(G9), can be considered desirable with low AUDPC values (547.94, 607.46, 621.38, 658.91, and 648.16 across seasons, respectively) and consistent performance over seasons with less than 20% CV. The ranking biplot highlighted Tapaswini (G32) with the highest AUDPC value with its nearness to the arrowhead and proximity to the center. In contrast, the more desirable genotypes (with low AUDPC values) were present near the proximity of IC 283139 (G19), Tetep (G28), IC 260917 (G4), IC 277274 (G10), and IC 277261 (G9).

#### Evaluation of the environment

**Figure 6:**
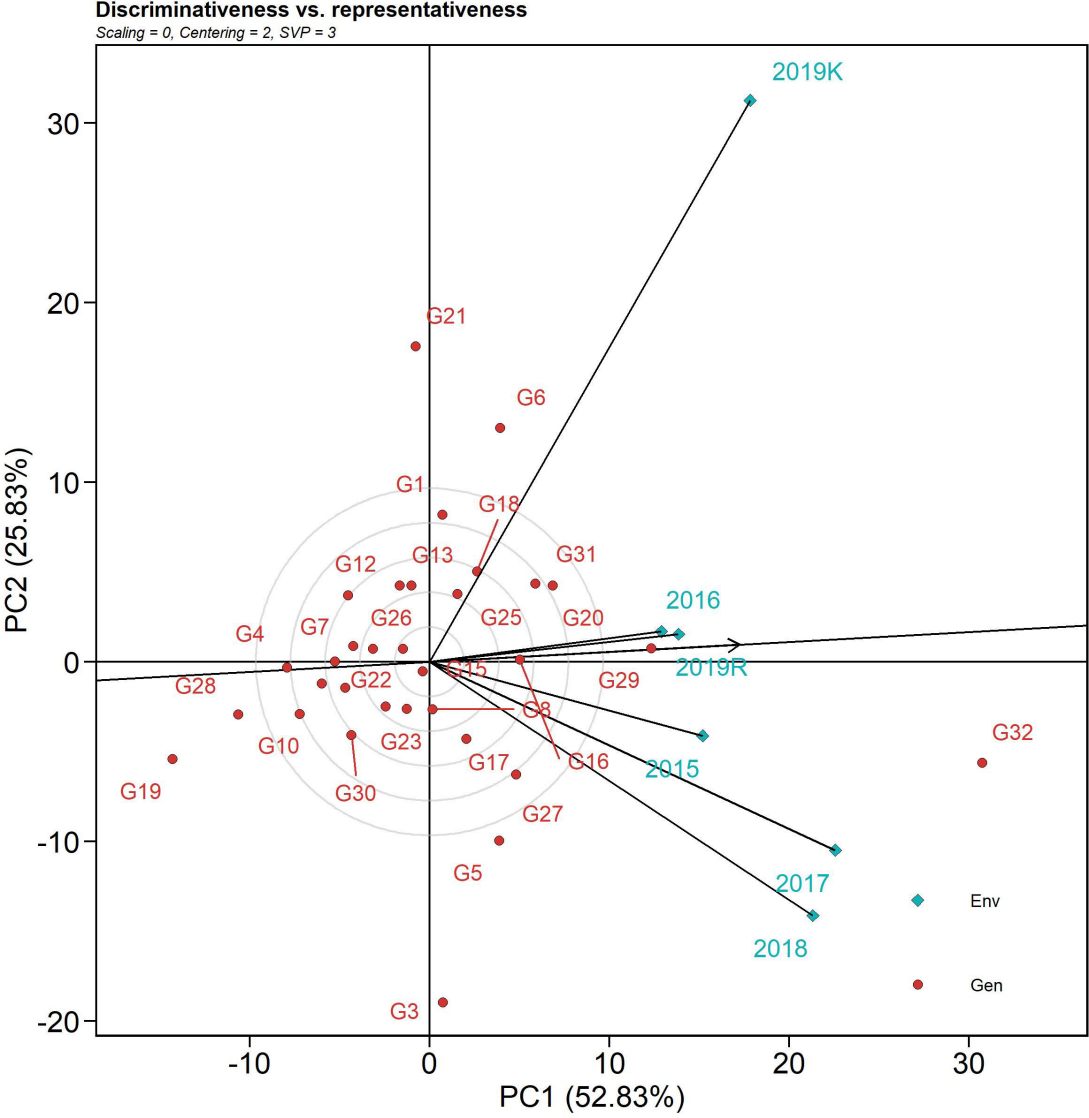
The discriminativeness vs. representativeness view of test locations is based on a GGE biplot of 32 rice genotypes across six seasons and two locations. The biplot was based on genotype-focused singular value partitioning (“SVP = 3”). Data were centered by means of the environments (centering = 2) and, therefore, are appropriate for visualizing the relationships among environments. This explained 78.6% of the total G + GE for the subset.

The discriminativeness (discrimination of genotypes) vs. representativeness (representing all the test environments) biplot tests the ideal test environment. This graph groups the test environment into three types: type I has short vectors providing little information regarding the genotypes and is not usually reliable as a test environment, type II has long vectors and short angles with the AEC abscissa and is, therefore, ideal for selecting superior genotypes, and type 3 has long vectors and large angles with the AEC abscissa. Among the six test seasons, 2019 *kharif* exhibited the longest environmental vector (large angle with the AEC abscissa), followed by 2018 and 2017 (**Figure 6**). In contrast, 2016 and 2019 *rabi* had medium-length projections. The AEC abscissa passing through the origin had a smaller angle with 2019 *rabi* and 2016, suggesting greater power of representation of the sheath blight reactions on the test genotypes. Hence, these two seasons can be regarded as representative test environments.

### Multi-trait stability index

**Figure 7:**
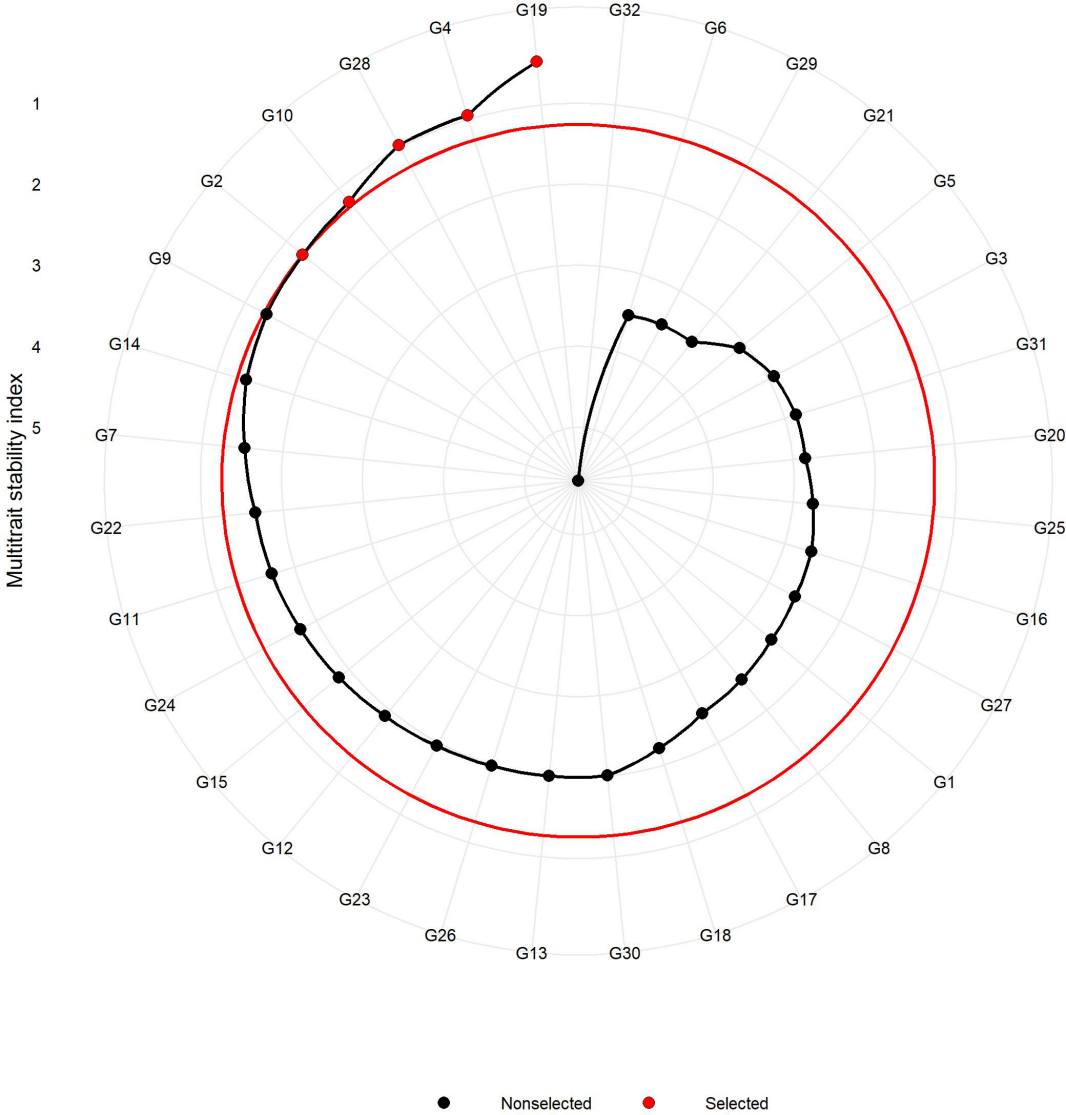
Genotypes are arranged based on a multi-trait stability index considering 15% selection intensity (the genotypes highlighted in red outside the inner circle are preferred for stability and ideal for selection).

The MTSI was employed to identify the stable resistant rice genotypes against sheath blight infection considering the multiple parameters in this study. The test for likelihood ratio indicated significant G x E interaction for all the studied traits except for PDI at 7 days (**Tables 5**, **6**), suggesting the influence of the variable effect of environments on the response of test genotypes. **Table 5** represents the factor analysis performed with the WAASBY index. The first component (FA1) had an eigenvalue of more than 1 and accounted for 82.30%. FA1 accommodated all of the six traits in this study and, after proper varimax rotation, the mean commonality (h) was 0.82. The selection differential for the WAASBY index was positive for all the traits under study, indicating that the study method was proficient in identifying stable genotypes with low disease reaction across six seasons. Selection differentials quantify a population’s mean trait value change between pre- and post-selection. The MTSI provided a positive selection differential for four studied traits. The selection differential ranged from 17.0 (PDI 28th day) to 22.9 (PDI 7th day). The mean of the selected genotypes (Xs) was higher than the original average (Xo). Genotypic values for the MTSI model with 15% selection intensity were estimated using the six traits (AUDPC, PDI 7th day, PDI 14th day, PDI 21st day, PDI 28th day, and PDI mean). The genotypes highlighted in red were selected as the ideal genotypes, as shown in **Figure 7**. Five genotypes in the following order, IC 256613 (G2), IC 277274 (G10), Tetep (G28), IC 260917 (G4), and IC 283139 (G19), were above the cut point. The genotypes IC 256613 (G2) and IC 277274 (G10) were plotted outside but closer to the red circle and, thus, are comparatively stable and more desirable for the traits under consideration.

**Table 5:** Selection differential of the WAASBY index for six sheath blight traits.

VAR		FA1	LRT		Communality	Uniquenesses	Xo	Xs	SD	SD%
			Gen	GxE						
1	AUDPC	-0.978	5.12e-12	5.77e-11	0.956	0.0445	67.3	86.4	19.1	28.4
2	PDI_7	-0.645	0.016	1.000	0.416	0.584	56.0	78.8	22.9	40.8
3	PDI_14	-0.942	5.20e-15	2.77e-03	0.887	0.113	74.8	92.6	17.9	23.9
4	PDI_21	-0.952	4.06e-11	2.12e-08	0.906	0.0938	60.9	81.8	20.9	34.3
5	PDI_28	-0.898	3.11e-14	4.10e-04	0.806	0.194	61.5	78.5	17.0	27.6
6	PDI_m	-0.984	1.43e-15	1.65e-07	0.967	0.0325	65.6	83.8	18.2	27.8
			Communality mean	0.823					

FA, factor analysis; X_o_, mean for WAASBY index of the original population; X_s_, mean for WAASBY index of the selected genotypes; SD, standard deviation.

**Table 6:** Selection gain (%) for the mean of six sheath blight traits.

	Traits	Factor	Xo	Xs	SD	SD%	h^2^	SG	SG%
1	AUDPC	FA1	749	594	-155	-20.7	0.808	-125	-16.7
2	PDI_7	FA1	12.1	11.1	-0.93	-7.7	0.455	-0.425	-3.5
3	PDI_14	FA1	18.0	13.0	-4.96	-27.6	0.842	-4.18	-23.3
4	PDI_21	FA1	31.7	25.2	-6.54	-20.6	0.795	-5.2	-16.4
5	PDI_28	FA1	42.2	33.4	-8.82	-20.9	0.834	-7.36	-17.4
6	PDI_m	FA1	25.9	20.7	-5.26	-20.3	0.848	-4.46	-17.2

FA, factor analysis; X_o_, mean for the trait of the original population; X_s_, mean for traits of the selected genotypes; SG, selection gain; SD, standard deviation; h^2^, heritability.

## Discussion

Sheath blight is the second most devastating fungal disease in rice, causing yield reductions of 20-50% depending on the severity of the infection (Mukherjee, 1978). It has recently grown to be a serious problem, particularly in areas of intensive rice farming. The primary causes of the dramatic increase in disease incidence are believed to be the monoculture of high-yielding semi-dwarf rice cultivars, high dosages of nitrogenous fertilizers, and the favorable microenvironment owing to the crop’s density (Cu et al., 1996). Although many cultural and chemical practices to control sheath blight are available, building inherent resistance in cultivated varieties can be a reliable solution for managing sheath blight disease in rice. The scope of this study was to evaluate the performance of 32 different rice genotypes across six seasons artificially inoculated with sheath blight pathogen *R. solani*, simultaneously evaluated in comparison with the performance of resistant (Tetep, Jasmine 85, and Teqing) and susceptible (Pusa Basmati-1 and Tapaswini) checks. This study’s findings could help farmers tackle sheath blight incidence with stable and tolerant varieties across seasons, especially in areas with intensive paddy cultivation. Such areas usually resort to monocropping and are often prone to sheath blight attacks owing to their sclerotia sustaining in the soil. In such situations, the only way out is to develop a tolerant variety with stable performance over seasons and environments.

Plant morphological traits such as plant height, flag-leaf length, flag-leaf width, tiller number, and panicle length followed a normal distribution. This establishes the wide range of trait expression present in the population under study and is, thus, a perfect group for genetic improvement studies. The skewness values for these traits also support a normal distribution. However, the kurtosis showed heavy-tailed values for plant height and tiller number. The coefficient of variation was considerable except for the tiller number. AUDPC had high kurtosis values for disease reaction-measuring traits, indicating heavy tails and a significant level of CV. The AUDPC values recorded in this study were significantly negatively correlated with the disease reaction.

**Figure 8:**
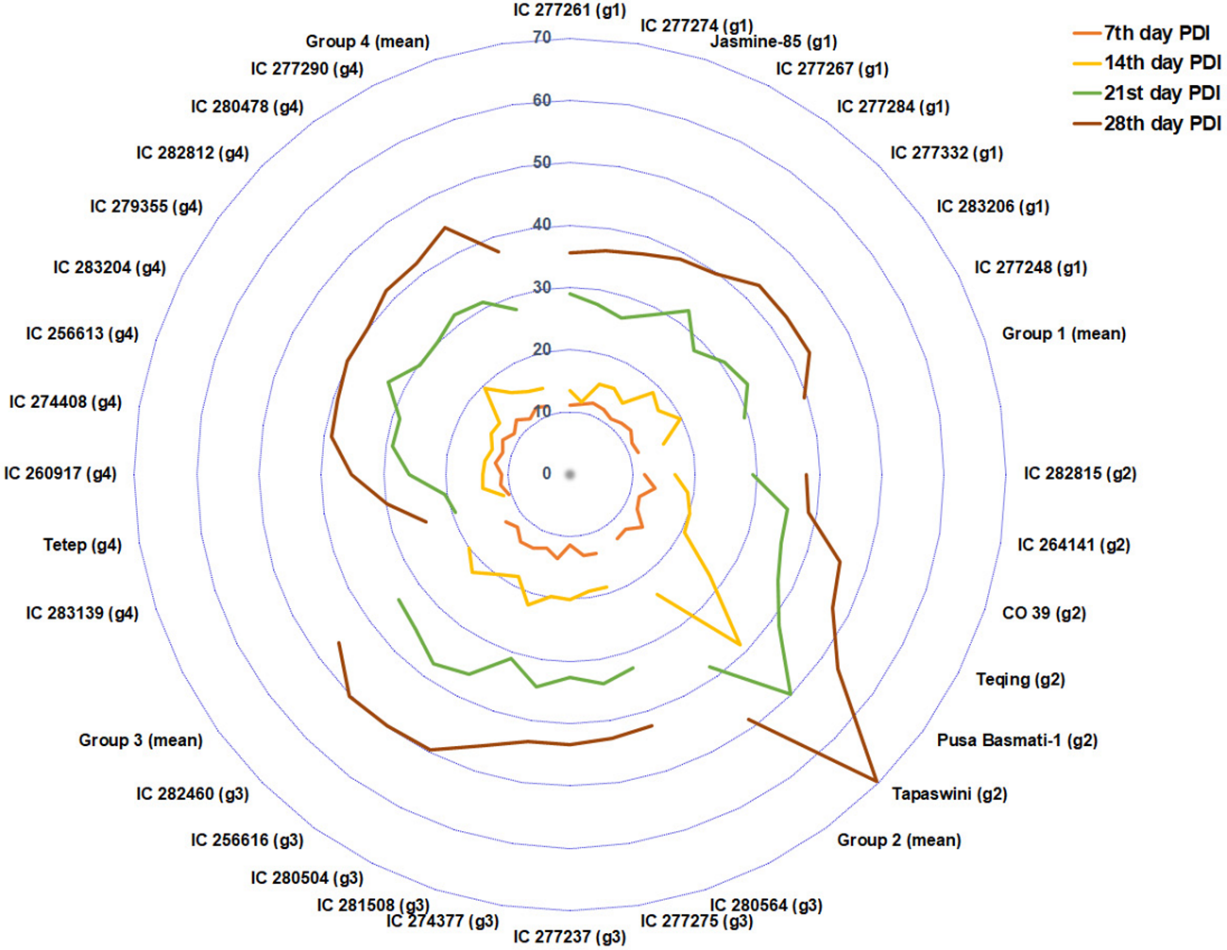
The PCA-based genotype grouping represented by PDI of 4 weeks. The PDI 28^th^ day of group 2 (susceptible genotypes) touches the outer ring, and group 4 exhibited a minimum PDI value.

PDI on the 7th day had significant negative associations with plant height, flag-leaf length, and flag-leaf width. This shows that initial infection and disease development depend on the shoot biomass accumulated in the plant via plant height, flag-leaf length, and flag-leaf width. However, the strength of this correlation decreases in successive weeks. This is because variations in the genotypic effect and physiological response triggering in different genotypes might have a significant role in the rate of disease reaction (Naveen kumar et al., 2022). The PCA biplot displayed a cumulative variation of 40.99% contributed by PC1 and PC2 in the population. The AUDPC, PDI 14th day, PDI 21st day, and PDI 28th day traits were clustered together, whereas PDI 7th day was separate. This indicates that the expression of disease reaction on the 7th day was preliminary and more uniform in all the genotypes, which might be due to the initial epiphytotic infection created artificially. However, the rate and intensity of disease spread operated variably for PDI 14th day, PDI 21st day, and PDI 28th day. The principal component analysis helped group the population into four clusters (**Figure 8**). Susceptible check Tapaswini was placed in a cluster opposite Tetep’s cluster. These groupings can help us to understand the trait expression associated with these contrasting lines in response to disease infection. Traits such as AUDPC, PDI 14th day, PDI 21st day, and PDI 28th day displayed higher expression in the cluster with Tapaswini, whereas their expression is low in the cluster with Tetep.

Weather parameters recorded on a daily basis across six seasons were correlated with the PDI mean value, and the pooled correlation results were studied. Maximum and minimum temperatures were negatively correlated with the PDI mean, whereas relative humidity was positively correlated with the PDI mean (**Supplementary Table 6**). Relative humidity is involved in the increased spread of the disease (Lenka et al., 2008; Shen et al., 2023). Although temperature is inversely proportional to relative humidity, the maximum and minimum temperatures are negatively correlated with the PDI mean. The conducive temperature for sheath blight infection is usually high, from 28 to 32°C, and relative humidity is approximately 97%, with disease infection being at its minimum, at 85% to 88% (Bhukal et al., 2015; Kaur et al., 2015).

### Stability models for G x E

There have been multiple studies involving stability analysis using different genotypes by environment datasets. The mean value of a trait is sufficient to study the stability of a genotype as long as there is an absence of G x E interaction (Yan and Kang, 2002). The main aim of a stability model is to identify a genotype by simultaneously considering the two sources of variation (G and G x E interaction) relevant to the mega-environment, genotype, and test environment evaluation (Yan et al., 2007). Multi-environment/season data may exhibit crossover types of G x E that indicate a change in the ranking of genotypes across the environment and non-crossover types of G x E that define a constant ranking of genotypes across the environment. Thus, the robustness of a model can be determined with the above checklist. AMMI and GGE biplots use these points and accurately represent G x E interaction. This has been used in multiple studies involving disease-resistant rice crops (Silva et al., 2011; Mukherjee et al., 2013; Persaud and Saravana Kumar, 2018). However, the reliability of AMMI analysis is considered higher or on par when compared to GGE biplots (Gauch, 2006; Anandan et al., 2009a). On the other hand, a newer approach, MTSI, has the advantage of simultaneously analyzing the stability and performance of multiple traits for test genotype selection as opposed to other stability analysis models that consider one character at a time. Additionally, when G x E interaction is high, it might affect selection efficiency; thus, a robust selection model using WAASM and MTSI is necessary (Abdelghany et al., 2021). This study is one of the first to analyze a dataset from six seasons for sheath blight resistance in rice across two diverse environments (Varanasi: 28.18° N, 38.03° E, and 75.5 masl, and Cuttack: 20°27’09” N, 85°55’57” E, 26 masl) via AMMI, GGE biplot, and MTSI model. In our investigation, partition, and interpretation were attempted using the AMMI stability technique, GGE biplot method, and MTSI. This study enabled the identification of a stable genotype across seasons for sheath blight resistance and, at the same time, presents a comparison of the results obtained from three different stability models.

### Additive main effects and multiplicative interaction

The AMMI model is one of the multivariate statistical techniques that aim to explore multi-directionality aspects and increase reliability. The significant sum of squares for genotypes demonstrated the diversity of the population genotypes under study, with differences in the genotypes accounting for the majority of the variability in disease infection response vis-à-vis the low sum of square values exhibited due to the environmental factor. This is in contrast to the findings of Anandan et al. (2009b); Mohammadi et al. (2018); Ngailo et al. (2019), and Bishwas et al. (2021), for which the environments displayed a greater and more significant sum of squares than the genotypes. This suggests the predominant contribution of genotypes over the AUDPC trait compared to the environment and G x E interaction factors. The large sum of squares for genotypes showed that the genotypes were diverse, and most variation in sheath blight resistance was due to significant differences among genotypic means. The effect of environment (E) was non-significant, implying that seasonal differences spanning the six test environments did not pose any significant difference in the expression of *R. solani*, causing sheath blight disease in the test genotypes. However, a significant role was played by the interaction between genotype and environment. In contrast, the mean AUDPC values calculated across seasons highlighted variation in their levels, indicating a possible role of G x E interaction.

In such studies, it is expected that test genotypes will have either a crossover type of G x E interaction, which shows variation in AUDPC values across seasons, or a non-crossover type of G x E interaction, showing constancy in their AUDPC values. The AMMI model partitioned this G x E interaction into five different principal components, of which the first three were significant, making major contributions for a total of 89.80% of the G x E interaction, implying that the first three PCs are enough to explain the G x E interaction effects of test rice landraces across six seasons.

The *AMMI 1 biplot* represents which-won-where information. The abscissa indicates the G and E effects, whereas its IPCA1 represents stability. Any displacement along the abscissa denotes an additive effect, whereas displacement in the ordinates denotes an interaction effect. The genotypes that are grouped together are assumed to be of similar adaptation. The genotypes with PC1 scores near zero indicate their suitability to all environments. The genotypes on the far right-hand side of the biplot represent high values of AUDPC (and hence are not desirable), whereas the ones on the left-hand side displayed lower disease reaction or low AUDPC values. Genotypes with IPCA values near zero are considered stable across seasons, whereas genotypes with higher IPCA scores, on either the negative or positive side, are suited only to the corresponding environment.

Additionally, a season and a genotype with the same sign have a positive interaction; when they are different, they show a negative relation (Lakew et al., 2017). Tapaswini (G32), the susceptible check, was on the far right-hand side with high values of AUDPC and was placed away from the origin. This shows that the line is highly susceptible and variable in its response across seasons. The genotypes with the lowest values of AUDPC were plotted at the right end of the x-axis: they were IC 283139 (G19) and Tetep (G28). In comparison with Tetep (G28), which had an AUDPC value of 547.94, IC 283139 (G19) performed better (479.69) than the susceptible check in sheath blight resistance but with a lower stability than Tetep (G28). However, the genotypes IC 256613 (G2), IC 260917 (G4), and IC 277261 (G9) had IPCA scores near zero and, thus, can be regarded as stable in their response toward sheath blight disease resistance, although the AUDPC values (658.91, 607.46, and 648.16, respectively) were higher than those of the resistant check. The *AMMI 2 biplot* is a graphical representation of the scores derived from PCs for explaining multi-environment G x E interactions. The AMMI 2 biplot accounted for 53% of the variation in PC1 and 23% of the variation in PC2. The genotypes plotted close to the ordinate express a general adaptation across seasons/environments, and those that are placed further from the origin are regarded as suitable for specific adaptation. Thus, the genotypes clustered around the origin are similar in their response toward all the environments. The environmental indicator with a shorter vector represents less G x E interaction and more stability. The plants with low AUDPC values and proximity to the origin can be regarded as stable and useful. All the seasons/environments were connected to the origin. Season 2015 had the shortest vector, thus exerting a weak interaction force, whereas 2019 *kharif* and 2016 exerted a strong interaction force. The genotypes present near the vertex are more responsive to specific environments. The two AMMI biplots helped in identifying the genotypes IC 256613 (G2), IC 277261 (G9), IC 260917 (G4), IC 277274 (G10), Tetep (G28), and IC 264141 (G5) as stable genotypes placed near the biplot origin and having low AUDPC values. These genotypes showed 1.19% and 0.72% increases in cellulose and hemicellulose, respectively, vis-à-vis susceptible cultivars. Conversely, susceptible cultivars contain 0.15% higher nitrogen content. The higher percentage of cellulose and hemicellulose with a lower amount of stem nitrogen might enhance the innate immunity of the resistant accessions. Previous reports documented that cellulose and hemicellulose of the host cell wall play a significant role in the level of resistance against *R. solani*, which degrades the host cell wall by cell wall degrading enzyme (CWDE), which is a physical barrier of the plant immune system (Zheng et al., 2013; Xia et al., 2017).

### GGE biplot

GGE biplot analysis doesn’t create a distinct partition between G and G x E; with this perspective, AMMI analysis is considered superior (Gauch, 2006). However, the GGE biplot can efficiently identify the G x E interaction pattern of the data and clearly illustrate which genotypes perform better in multiple test environments compared to AMMI (Yan et al., 2000).

The *which-won-where* polygon includes the furthest points from the origin as its corners such that it accommodates all other data points within the figure. The perpendiculars drawn from the origin to each side of the polygon separate the biplot into several vectors, with one genotype (located on the vertex) performing the best in the respective season. The equality lines divided the biplot into four sectors and environmental indices were placed in two of these sectors. The genotypes placed under the respective environments performed best in those sectors, while the genotypes placed in a section of the biplot where there was no environmental indicator indicated poorly performing genotypes with respect to stability. This biplot helped in identifying genotypes with high disease resistance across environments. The susceptible genotypes with high AUDPC values were placed in the sector with Tapaswini (susceptible check) (except 2019 *kharif*) but were not desirable. The resistant check was placed in the opposite sector without an environment index; thus, it can be considered poor in stability. However, a few genotypes, such as IC 260917(G4), IC 277274(G10), and IC 256613(G2), were found to have comparatively low AUDPC values and were placed nearer the origin, proving their stability. The *mean vs. stability* analysis plots two straight lines: a vertical AEC abscissa and a horizontal AEC ordinate. The single arrow-headed line points toward the greater mean performance and, thus, the genotypes placed behind the arrow were found to have low AUDPC values (higher resistance). The vertical abscissa determines the stability of the genotypes such that a genotype with zero projection from the horizontal axis is the most stable. Accordingly, Tetep (G28), IC 260917 (G4), IC 277274 (G10), IC 256613 (G2), and IC 277261 (G9) were identified as desirable genotypes for further improvement as they had lower AUDPC values and minimal projection from the horizontal axis. *Representativeness and discriminativeness* biplots define the best-suited environment for the test genotypes. The test environment is better when the angle between the AEC abscissa and the test environment vector is less than when bigger angles are generated. An arrow indicates the direction of the AEC abscissa line and the average value of the test environment is indicated by a small concentric circle, with the discriminating ability being inferred from the length of the test environment vector (Bishwas et al., 2021). However, discriminativeness (the capacity of an environment to differentiate genotypes) and representativeness (the capacity of an environment to represent all other examined settings) are two characteristics that indicate how good the tested environments were (Oladosu et al., 2017). Based on the angle between the vector and AEC abscissa as well as the length of the vector, 2016 and 2019 *rabi* can be regarded as environments that can ideally discriminate genotypes on the basis of their reaction against sheath blight infection. The temperature range during 2016 was 25.6-31.9°C and the relative humidity was 77.7-87.3%. Similarly, the temperature range during the 2019 *rabi* was 25.2-36.6°C, as the temperature was high there, and the humidity was in the range of 60.5-87.4%. In both seasons, the initial AUDPC values were greatly influenced by relative humidity and temperature range, thus establishing the role of G x E interaction. The PDI mean varied from 11.3 to 12.9 on the 7th day after infection. The highest PDI mean was 12.9 in 2019 *kharif*, with the highest RH (94.6%) across all seasons and one of the lowest active sunshine hours (5.1 h). The AUDPC value on the 28th day for 2016 was 48.2 and for 2019 *rabi* was 43.6, with relative humidity ranging from 85.1% (evening) to 91.4% (morning) and 60.6% (evening) to 89.1% (morning), respectively. Additionally, the number of sunshine hours in 2016 was 4.2 h, whereas in 2019 *rabi*, it was 6.3 h. The difference in AUDPC values in these two test locations clearly shows them to be influenced by relative humidity (Lenka et al., 2008; Thind et al., 2008; Biswas et al., 2011; Pin et al., 2012) and active sunshine hours. These weather regimes can play a substantial role in disease establishment and spread and can consequently reveal the true genetic potential of test genotypes. The study locations themselves were significant factors accounting for total variation; similar results were also reported by Krishnamurthy et al. (2021).

#### Multi-trait stability index

Selection procedures in crop improvement programs involve simultaneously improving multiple traits and evaluating the stability of such traits across seasons/environments. However, stability models such as AMMI, Eberhart, Russell, etc., study G x E interaction by treating each trait separately, as a result, the effectiveness of determining a stable genotype across traits and environments is reduced. The MTSI is a rather new approach that aids in identifying a superior genotype with respect to multiple traits at the same time and is more in use in recent times for various crops (Broich and Palmer, 1980; Hussain et al., 2021; Koundinya et al., 2021; Lima et al., 2022; Yue et al., 2022). It uses the genotype-ideotype distance (Euclidean) using factor analysis scores (Olivoto and Lúcio, 2020). The MTSI is predicated on the genotype-ideotype distance projected with factor analysis values. The most stable genotypes with low AUDPC values for sheath blight (i.e., negative selection differentials for AUDPC that needed to be reduced) were revealed using the MTSI method. Given that it provides a robust and easy-to-comprehend selection method, the MTSI might be useful for plant breeders in selecting genotypes based on multiple traits. The genotypes with lower values of MTSI are the ones with higher stability based on the traits under consideration. In our investigation, five traits were considered for MTSI analysis: PDI 7th day, PDI 14th day, PDI 21st day, PDI 28th day, and PDI mean. Genotypes IC 256613 (G2), IC 277274 (G10), Tetep (G28), IC 260917 (G4), and IC 283139 (G19) were considered the most desirable.

Compared with the susceptible check, the identified stable lines had low stem nitrogen and high hemicellulose content, which might contribute to the low AUDPC values in the identified lines with stability against sheath blight. Previous reports have confirmed that high doses of nitrogenous fertilizer application have led to increased infection, but decreasing the dose can hamper plant growth and yield. Thus, the search must be for a genotype with higher nitrogen use efficiency instead of accumulating nitrogen in the stem. In this context, reports of ammonium transporters (belonging to the *OsAMT* family) suggest that these transporters have helped increase nitrogen assimilation and also have a role against sheath blight in rice, thereby aiding both growth and defense (Pastor et al., 2014; Wu et al., 2022). Similarly, hemicellulose has a role in plant disease development by regulating cell wall composition (mainly xyloglucan and xylan). Out of these four genotypes, IC 283139 has been earlier identified as a promising line for sheath blight resistance, and biochemical analyses have reported the presence of higher polyphenol oxidase, peroxidase, total phenol, phenylalanine ammonia-lyase, catalase, and superoxide dismutase in resistant lines vis-à-vis susceptible ones (Naveen kumar et al., 2022). Similarly to our previous study, we reported that these resistant genotypes showed more defense (peroxidase, polyphenol oxidase, phenylalanine ammonia-lyase, and total phenol) and antioxidant (catalase and superoxide dismutase) enzymes than the susceptible genotypes (Naveen kumar et al., 2022). These enzymes might have enhanced the innate immunity of these resistant genotypes. Our results were corroborated by those of Bindschedler et al. (2006) and Marjamaa et al. (2009), who reported that peroxidases can function to maintain the level of H_2_O_2_ and play a major role in cell wall regeneration and thickening.

## Conclusions

Rice not only satisfies the nutritional demand of most Asian countries but also sustains their economic well-being. The changing climate and life cycle of many pathogens negatively impact the crop’s growth and yield. Sheath blight is one of the deadliest diseases in rice, and limited work has been reported to date concerning GxE interaction. This paper has identified sheath blight tolerant genotypes simultaneously, compared the different stability models, and employed a recently developed MTSI model. The common genotypes that were identified as stable across six seasons in the three other stability models were IC 283139 (G19), Tetep (G28), IC 260917 (G4), and IC 277274 (G10), with AUDPC values of 658.91, 607.46, 479.69, and 547.94, respectively. Closer study of the qualitative traits of these genotypes revealed that nitrogen content (Savary et al., 1995; Slaton et al., 2003; Schurt et al., 2016) and hemicellulose (Hapsari and Poromarto, 2020) might have played a role in imparting resistance in the above genotypes. This study has helped us identify promising stable genotypes with low disease infection over six seasons and across two locations. In the future, the identified stable genotypes could be valuable in analyzing resistance mechanisms and their impact on grain yield.

## Data availability statement

The original contributions presented in the study are included in the article/**Supplementary Material**. Further inquiries can be directed to the corresponding authors.

## Author contributions

SP: Writing – review & editing, Conceptualization, Formal Analysis, Investigation, Methodology, Writing – original draft. NR: Conceptualization, Formal Analysis, Investigation, Methodology, Writing – original draft. LP: Conceptualization, Formal Analysis, Investigation, Writing – review & editing. GS: Formal Analysis, Investigation, Methodology, Writing – original draft. PS: Methodology, Writing – review & editing. RS: Methodology, Writing – review & editing. PV: Methodology, Writing – review & editing. HS: Writing – review & editing, Methodology. AM: Writing – review & editing. AA: Conceptualization, Investigation, Project administration, Supervision, Writing – original draft, Writing – review & editing. JA: Funding acquisition, Writing – review & editing.
